# Risk of hospitalization associated with different constellations of home & community based services

**DOI:** 10.1186/s12877-022-03676-2

**Published:** 2023-01-20

**Authors:** Raymond Van Cleve, Evan Cole, Howard B. Degenholtz

**Affiliations:** 1 Big Data Science Training Enhancement Program Fellow, Center for Innovation to Implementation , VA Palo Alto Health Care System, CA Palo Alto, USA; 2 Post Doctoral, Stanford University, CA 94305 California, USA; 3 Department of Health Policy and Management, Medicaid Research Center, University of Pittsburgh, Pittsburgh, USA

**Keywords:** Home and Community Based Services, Acute Inpatient hospitalization, Medicaid

## Abstract

**Background:**

Identify the association between specific combinations of home and community-based services (HCBS) and risk of acute hospitalization.

**Methods:**

Data for this study came from Pennsylvania Medicaid claims and Medicare records. This was a retrospective, observational cohort study that examined hospitalization, HCBS service use and patient characteristics between July, 2014 and December, 2016. This analysis compared risk of inpatient hospitalization risk for community dwelling disabled older adults using a range of Medicaid financed HCBS. Twelve constellations of HCBS were identified representing different combinations of common services (personal assistive services [PAS], delivered meals, and adult day care). Since HCBS users are not randomly assigned to different combinations of services, we used logistic regression to estimate the predicted probability of experiencing hospitalization conditional on the constellation of services, and adjusting for demographics, health and level of disability.

**Results:**

The most common constellation was people who used under four hours of PAS per person per day. This group experienced a hospitalization rate of 13.7%. however, those individuals receiving more than 4 h per person per day experienced only a 10.2% hospitalization rate. Similar trends were seen for people who used PAS in combination with home delivered meals. However, those who used adult day care experienced higher hospitalization rates as the number of hours of personal assistive service increased: increasing from 6.8% among those with under 4 h, to 8.6% among those with 8 or more hours per person per day.

**Conclusion:**

Using medium and high levels of PAS was associated with lower hospitalization risk for people who PAS alone or in combination with delivered meals. By contrast, higher levels of PAS was associated with increased hospitalization for adult day users (both alone or in combination). Policy makers should consider offering higher levels of PAS to offset potential risk of hospitalization. Future research is needed to explain the association between adult day care and risk.

**Supplementary Information:**

The online version contains supplementary material available at 10.1186/s12877-022-03676-2.

## Introduction

America’s aging population poses a challenge never before encountered [1–3]. Ideally older Americans can continue to live independently and have their individual needs and limitations can be met, rather than living in a nursing home [4, 5]. This system of care must also be financed so costs do not become unmanageable, especially for the public payers like Medicare and Medicaid [6, 7]. One proposed strategy to provide high-quality long-term services and supports (LTSS) sustainably is to shift care more towards home and community-based services (HCBS). HCBS can be designed to support an individual’s unique needs [4, 8–10]. HCBS is also much less expensive than skilled nursing facilities [7].

The scope of HCBS is to support a person’s functional needs and everyday living. The in home services that aid people are not staffed by trained clinicians and are not designed to provide medical care. A potential problem with only providing functional support is that some health needs may go unaddressed, causing HCBS recipients to have an increased risk of hospitalization, a finding of previous research [11–13]. A hospital admission may lead to a permanent nursing home placement [14–16]. If HCBS is not adequately addressing the risk of hospitalization, then it is not supporting people to live independently nor is it lowering costs of care for these individuals [17].

Previous research on HCBS service lines has examines outcomes associated with individual services. Attendant care or personal assistive services (PAS) is commonly used in home services [18–20]. PAS has been shown to protect against hospitalizations as well as support a person’s autonomy [21–23]. Two studies focused on nutrition for elderly people found that providing delivered meals to HCBS beneficiaries decreased their likelihood for admission to a nursing home and supported independent living [24, 25]. Studies have demonstrated that adult day care is associated with improved psychological outcomes [26].

This study addresses this research gap by examining the risk of hospitalization associated with the different constellations of HCBS within the Pennsylvania 1915(c) Medicaid Waiver. This study will identify what services in an HCBS program can complement each other by providing additional protection from going to the hospital, or if specific services are more effective when administered at higher levels.

## Methods

This study examined how the type and amount of HCBS is associated with risk of experiencing an acute inpatient hospitalization among disabled Pennsylvanians aged 65 and older. We used Medicaid and Medicare claims and individual assessment data to measure physical function and other relevant risk factors for subsequent hospitalization over a 30-month time period. The analytic data set, described in detail below, was constructed to establish a chronological sequence between the main independent variable (HCBS), covariates, and the outcome of interest (hospitalization). Due to the observational nature of the data, we do not advance a causal interpretation of the findings.

### Sample

The data for this study came from the Pennsylvania Department of Human Services. The unit of observation was the person quarter [11]. The earliest a person can be observed in our data is July of 2014 and the latest a person could be observed in our data is December of 2016. To be counted as receiving HCBS the person needed to be enrolled in the waiver for the whole quarter and receive some HCBS during that quarter. The sample was limited to people enrolled in traditional fee-for-service Medicare, since hospitalization claims were not available for people enrolled in Medicare Advantage.

### Covariates

Comprehensive assessment data from the Pennsylvania Department of Aging was used to construct measures of physical and cognitive function. The comprehensive assessment is used to determine if an individual is eligible for Medicaid HCBS Waiver services and is repeated annually or if there is a change in the participant’s health or functional status has changed. From these data we extracted measurements of limitations in basic activities of daily living (ADL; eating, bathing, toileting, transfer, walking indoors, and dressing), instrumental activities of daily living (IADL; housework, walking outside, managing money, using a telephone, preparing meals, and shopping), and continence (bladder and bowel control, ability to manage an ostomy bag). Each participant was rated as totally independent, requiring some assistance, or totally dependent on each task, and each task was assigned a weight based on a magnitude estimation score and then converted into zero to ten scales for ADL, IADL, and continence [27, 28]. A zero indicates no limitation and a 10 indicates complete assistance required for all aspects of each domain. We also included indicators of Parkinson’s disease and stroke (based on self or proxy report); conditions which are associated with significant levels of dependence that may not be captured by IADL and ADL measures. Finally, since the assessment instrument does not include a reliable measure of cognition, we include an indicator of Alzheimer’s Disease or other form of Dementia (ADRD) as noted in the assessment (based on self or proxy report).

Medicaid and Medicare claims data were used to construct indicators of 27 chronic diseases [29]. Based on the distribution of the number of chronic conditions per person, the count was categorized as zero or one condition, two or three conditions, 4 or five conditions, or six or more.

Race and ethnicity were coded as non-exclusive categories of non-Hispanic black, non-Hispanic white, non-Hispanic Asian, Hispanic, or other. Urbanicity was defined using the National Center for Health Statistics Urban-Rural Continuum Codes (RUCC) that classifies counties based on their population and their proximity to an economic center [30].

### Main independent variable

The main independent variable was use of HCBS services. We focused on three specific services that were used most frequently and are important components of all HCBS programs [31]: PAS, Home Delivered Meals, and Adult Day Care. These services are billed on a per encounter basis. PAS was billed in 15-minute increments; we calculated the average minutes per day per quarter. Home delivered meals and adult day care are billed per meal or per day (respectively). Due to the distribution of these data (low prevalence), these were covered to binary measurements, indicating if that person any home delivered meals or adult day care during the person quarter.

The service central to many HCBS programs is PAS [19, 20] and 97% of the people in our analysis regularly used some sort of personal assistive services. The daily average PAS ranged from one hour to 24 h, however the distribution was skewed with few people having relatively high levels of PAS. Previous research found that the mean hours of personal care per day for people with 3 ADL limitation is about 4 h; people with 3 or more ADL limitations use an average of about 8 h per day [32]. We therefore classified PAS use per day as low (up to 4 h), medium (4 to 8 h), and high (more than 8 h) PAS users.

We constructed 12 constellations of HCBS based on combinations of the three most common services: PAS, adult day care, and home delivered meals. This 12 constellations are: (1) low levels of only PAS, (2) low levels of PAS and any adult day care, (3) low levels of PAS and any home delivered meals, (4) low levels of PAS, any adult day care, and any home delivered meals, (5) medium levels of only PAS, (6) medium levels of PAS and any adult day care, (7) medium levels of PAS and any home delivered meals, (8) medium levels of PAS, any adult day care, and any home delivered meals, (9) high levels of only PAS, (10) high levels of PAS and any adult day care, 11) high levels of PAS and any home delivered meals, 12) high levels of PAS, any adult day care, and any home delivered meals.

### Outcome variable

The outcome of interest was risk for experiencing an acute inpatient hospitalization during the person-quarter. Data on this came both from Medicaid and Medicare claims. This was a binary variable indicating if a person had or had not experienced a hospitalization during that quarter.

### Analysis

We used logistic regression to estimate the association between hospitalization and selected constellations of HCBS. The results are presented as predicted probabilities to facilitate meaningful comparisons of the different services.

Included in this analysis was a reference group of community dwelling elderly people who had applied to receive HCBS but had been deemed ineligible for Medicaid funded HCBS. These people were deemed ineligible because they did not meet the statutory requirement for waiver services of needing nursing home level of care. However, since this group applied for waiver services, they are a potential control for self-selection of waiver participants. Adjusting for functional status, this group is similar to low-acuity waiver participants (i.e., they are just below the threshold for nursing home level of care). By comparing waiver users to this population, we hypothesize that we can estimate the benefit of using any HCBS compared to a similar population of people not receiving any. If people using HCBS have a significantly lower risk of hospitalization than people less disabled living in the community and not receiving HCBS, this suggests HCBS is providing some benefit at the threshold.

Descriptive, bivariate analysis was conducted to examine the characteristics of the sample of participants and the association between physical function and use of HCBS. Multivariate logistic regression was used to estimate the probability of experiencing a hospitalization for people in each HCBS constellation. Covariates in the model were race, gender, age, rurality, location within the state of Pennsylvania, urinary and fecal continence, the person’s ADL and IADL levels, living arrangement, the number of chronic conditions, and the number of quarters the person had been observed in our data. Since individuals could appear in the data set for multiple quarters, standard errors were clustered at the person level [33].

### Sensitivity analysis

Alternative models were estimated to examine whether the association between risk of hospitalization and constellation of HCBS was robust to assumptions about the relevant comparison group and the estimation strategy. The first alternative model run was a model that excluded people who were not using any HCBS (i.e., not Waiver eligible). This model used people receiving only low levels of PAS as the reference group. The second alternative specification used generalized estimating equation (gee) with logit link and robust standard errors as an alternate approach to adjusting for repeated observations. These alternative specifications are included as an online appendix.

## Results

Assessment records for total of 21,818 older adults were matched to Medicaid claims and enrollment files for the period between July 2014 and December 2016. After excluding 1,661 (7.6%) individuals with missing data on one or more variables, the final analytic file consisted of 12,973 Medicaid 1915(c) waiver participants and 7,184 Medicaid participants who had a comprehensive assessment, but were not eligible for waiver services. The sample represented 142,416 person-quarters (mean 7.1 quarters per person).

The descriptive statistics show that the majority of the people in our sample were female, white, and lived in urban areas (See Table 1). The average age of the people receiving HCBS was 79.3 (9.3) and the average age of the community dwelling elderly people not receiving HCBS was 75.8 (8.6) (see Table 1). People receiving HCBS had a 3.04 higher ADL level (*p* < 0.001), a 2.62 IADL level (*p* < 0.001), and a 2.08 higher level of incontinence than those not enrolled in HCBS (*p* < 0.001) (see Table 1). The overall hospitalization rate (by person-quarter) was 13.36%; 11.14% of person-quarters without any HCBS had at least one hospitalization compared to 14.7% of person-quarters with any HCBS (chi square 393.29; *p* = 0.000).

**Table 1:** Demographic Characteristics of HCBS Users and non-Users

		PAS Hours Per Day			
	No HCBS (*n* = 6,736)	< 4 h (*n* = 6,812)	> 4 and < = 8 (*n* = 3,706)	> 8 (*n* = 790)	Total (*n* = 18,071)
Age					
65–69	37.35	23.26	22.23	13.4	22.8
70–74	21.17	16.8	14.15	12.01	15.9
75–79	17.13	18.27	15.6	15.68	16.41
80–84	11.67	17.19	17.35	18.2	16.18
85 and Older	12.68	24.48	30.67	40.71	28.72
Gender (Female)	67.81	70.93	76.36	80.56	70.38
Rural	16.21	12.74	14.38	19.82	15.14
Race					
Non-Hispanic White	59.69	47.98	56.72	67.13	67.05
Non-Hispanic Black	19.87	27.67	25.98	17.77	19.12
Asian	8.3	7.53	8.34	7.23	6.08
Hispanic	1.55	0.95	1.38	1.27	1.19
Other	10.6	5.87	7.58	6.6	6.56
Living Arrangement (Lives Alone)					
Spouse	13.82	14.67	9.87	7.97	9.87
Child	12.91	17.91	30.49	27.72	15.06
Other Relative	12.78	11.51	14.80	15.57	10.16
Other Person	19.50	9.29	10.75	11.39	28.28
Chronic Conditions					
0–1	30.43	18.53	17.98	26.84	23.22
2–3	27.97	28.26	27.52	28.86	28.02
4–5	22.78	24.74	23.91	21.01	23.67
6 or More	18.82	28.48	30.59	23.29	25.08
Continence (0–10)	2.16 (2.82)	3.85 (3.29)	5.25 (3.54)	6.63 (3.34)	3.62 (3.44)
Basic ADL (0–10)	3.61 (2.94)	6.30 (2.43)	7.61 (1.95)	8.61 (1.47)	6.6 (3.05)
Instrumental ADL (0–10)	4.44 (2.5)	5.8 (2.05)	6.84 (1.91)	7.96 (1.55)	5.58 (2.42)
Alzheimer’s Disease or Related Dementia	15.24	22.20	33.69	51.27	23.23

All bivariate and group comparisons are statistically significant

More than one third of the sample (37.8%) used low levels of PAS (Table 1). People with either medium or high amounts of PAS made up 24.9% of the sample. Community dwelling elderly people not receiving any Medicaid funded HCBS were 37.3% of the sample. Table 2 presents the distribution of person-quarters by combinations of HCBS service. About 22.8% of person-quarters had any home delivered meals, while only 10.2% had any use of adult day care.

**Table 2:** Number of Person-Quarters with Combination of HCBS

Level of PAS (Hours per day)	PAS Only		PAS and Adult Day		PAS and Meals		Pas and Adult Day and Meals	
	n	%	n	%	n	%	n	%
< 4	31,911	18.77%	7,404	4.36%	14,160	8.33%	617	0.36%
>=4 and < 8	27,319	16%	2,772	1.63%	7,282	4.28%	302	0.18%
>= 8	8,370	4.92%	233	0.14%	1,843	1.08%	77	0.05%

**Fig. 1:**
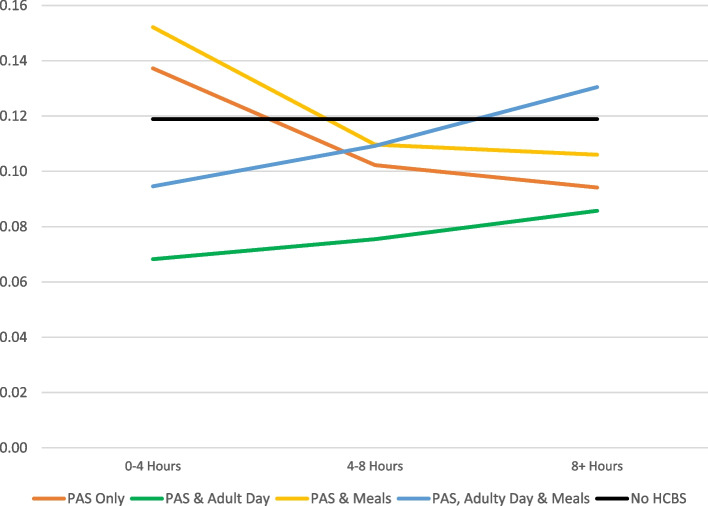
Predicted Probability of Experiencing a Hospitalization by Type and Amount of HCBS

Community dwelling elderly people not receiving any Medicaid funded HCBS had an unadjusted hospitalization rate of 11%. People receiving HCBS had a 14% rate of hospitalization. The multivariate logistic regression results are presented on Table 3; predicted probabilities were generated for selected sub-groups of people to elucidate the findings, and are discussed below and presented in Fig. 1.

**Table 3:** Association Between All-Cause Hospitalization and Type and Amount of HCBS

	Odds Ratio	95% Confidence Interval	
Type and Amount of HCBS (ref. No-HCBS)			
Low PAS			
Only PAS	1.18	1.13	1.23
PAS and Adult Day Care	0.54	0.49	0.6
PAS and Delivered Meals	1.33	1.26	1.41
PAS, Adult Day Care, and Delivered Meals	0.77	0.59	1.01
Medium PAS			
Only PAS	0.84	0.8	0.89
PAS and Adult Day Care	0.6	0.53	0.7
PAS and Delivered Meals	0.91	0.84	0.99
PAS, Adult Day Care, and Delivered Meals	0.91	0.64	1.28
High PAS			
Only PAS	0.77	0.71	0.84
PAS and Adult Day Care	0.69	0.46	1.05
PAS and Delivered Meals	0.88	0.76	1.02
PAS, Adult Day Care, and Delivered Meals	1.11	0.58	2.14
Age (ref. 65–69)			
70–74	0.96	0.91	1.01
75–79	0.91	0.87	0.96
80–84	0.93	0.88	0.98
85 and Older	0.99	0.94	1.04
Gender (ref. Female)	0.88	0.85	0.91
Rural	1.10	1.05	1.16
Race (ref. Non-Hispanic White)			
Non-Hispanic Black	1.13	1.08	1.17
Asian	0.56	0.52	0.61
Hispanic	1.00	0.88	1.14
Other	0.81	0.75	0.86
Living Arrangement (ref. Lives Alone)			
Spouse	0.80	0.75	0.84
Child	0.98	0.94	1.03
Other Relative	0.99	0.94	1.04
Other Person	1.06	1.01	1.12
Chronic Conditions (ref. 0–1)			
2–3	1.14	1.08	1.2
4–5	1.64	1.55	1.73
6 or More	3.41	3.25	3.59
Continence (range 0–10)	1.02	1.01	1.02
Basic ADL (range 0–10)	1.04	1.03	1.04
Instrumental ADL (range 0–10)	1.07	1.06	1.08
Alzheimer’s Disease or Other Dementia	0.91	0.88	0.95
Time in program (months)	1.02	1.02	1.03

*HCBS* Home and Community Based Services, *PAS* Personal Attendant Services, *ADL* Activities of Daily Living

After adjusting for health, disability, demographic factors, community dwelling duals receiving no Medicaid funded HCBS faced an 11.6% (95% CI:11.3%, 11.9%) probability of experiencing a hospitalization (see Fig. 1). The predicted probabilities of hospitalization for people using HCBS ranged from 15.1 to 7.5% depending on a person’s constellation of HCBS (see Fig. 1).

Holding all other variables at their averages, people using only low levels (0<-4 h) of PAS faced a 13.9% probability (95% CI:13.5%, 14.3%) of experiencing hospitalization. People using only medium (4<-8 h) levels of PAS faced a 10.2% probability (95% CI: 9.8%, 10.6%) of experiencing a hospitalization. People using only high levels of PAS (8 + hours) face a 9.5% (95% CI: 8.8%, 10.1%) probability of experiencing a hospitalization (see Fig. 1).

People using low levels of PAS and adult day care experienced a 7.5% probability (95% CI: 6.8%, 8.1%) of hospitalization. People using medium levels of PAS and adult day care faced an 8.2% probability (95% CI: 7.2%, 9.2%) of experiencing a hospitalization. People using high levels of PAS and adult day care faced a 9.4% probability (95% CI: 5.9%, 12.9%) of experiencing a hospitalization (see Fig. 1).

People using low levels of PAS and home delivered meals experienced a 15.1% probability (95% CI: 14.5%, 15.7%) of hospitalization. People using medium levels of PAS and home delivered meals faced a 10.7% probability (95% CI: 10.0%, 11.4%) of experiencing a hospitalization. People using high levels of PAS and home delivered meals faced a 10.2% probability (95% CI: 8.9%, 11.5%) of experiencing a hospitalization (see Fig. 1).

People using low levels of PAS and both adult day care and home delivered meals faced a 10.1% probability (95% CI: 7.7%, 12.4%) of experiencing a hospitalization. People using medium levels of PAs, adult day care, and home delivered meals faced an 11.1% probability (95% CI: 7.7%, 14.4%) of experiencing a hospitalization. People using high PAS, home delivered meals, and adult day care faced a 13.2% probability (95% CI: 5.7%, 20.8%) of experiencing a hospitalization (see Fig. 1).

## Discussion

The first takeaway from this analysis is that people using high and medium levels of PAS face a lower risk of hospitalization than the community dwelling elderly people with no HCBS as well as people using less than four hours of PAS per day. The most common constellations of HCBS are those the least intensive in terms of PAS, suggesting that a sizeable fraction of HCBS users may be using the bare minimum amount of HCBS when using more hours of PAS or PAS in combination with other services might be more appropriate to support their needs. Although it is beyond the scope of our data, a likely mechanism is that greater time in the home is required to assure adequate nutrition and hydration, medication adherence, and to observe subtle changes in health that can be addressed early.

The second takeaway is that people receiving home delivered meals and PAS were at a slightly higher risk of hospitalization than people only receiving PAS. This was noteworthy because previous literature has found that providing a meal can prevent or delay a person’s risk of nursing home placement [24, 25]. One potential cause of this elevated risk is that people who receive a home delivered meal are more likely to live alone and experience loneliness and isolation [34]. These individuals may not be managing their health needs or engaging a support system in an appropriate manner. Our data was limited in that the only measurement of isolation available to us was a person’s living situation. Some people who live alone may have family who live very close or may have a tight social network that can support them. Others may not have any form of social support. Understanding the degrees of isolation people face and what services may be able to address that isolation is an important future research topic.

We conducted a sensitivity analysis excluding people not using HCBS (see Appendix). This model still shows the same relationship between level of PAS and hospitalization, and the same patterns with respect to adult day care and home delivered meals. Finally, the results are not sensitive to the choice of estimators. A second sensitivity analysis using Generalized Estimating Equations (See Appendix), finds the same pattern of results. The general estimating equation also used any hospitalization during the person quarter as the outcome variable.

### Limitations and future research

The primary limitation of this study is that it is an observational study that relies on actual service use, rather than randomized assignment to the type and amount of HCBS. Such a study would be prohibitively expensive, complex and would raise serious ethical and equity concerns. Thus, we were limited to identifying groups of people using specific combinations services and their associated risk for experiencing a hospitalization. Some combinations of HCBS services were rare, leading to low statistical power for some comparisons. However, the main findings regarding the amount of PAS are based on stable estimates.

We also did not look at the total array of services offered to each client. We did not have information in our data to see specific services, for example we did not have any way of measuring or examining the types of home modifications people received. We also were not able to see a person’s use of transportation services (i.e. did a person use subsidized public transit, did a person use a service like access, did a person have their attendant aid provide transportation). We also did not observe family care and the presence of a family caregiver not living with the person. We also did not observe tasks the personal care attendant was doing. In some instances, the personal care attendant might be cooking and in other instances a person may just be getting a meal to give the attendant more time to focus on other things. Ideally for a future study, we could have greater insight into services a person was receiving and what the personal care aid was doing beyond just what was being is billed.

## Conclusion

We found that the type and amount of PAS is associated with risk of acute hospitalization. This suggests that HCBS users with relatively low levels of PAS may benefit from additional units of service through reduction of hospitalization rates. As policy makers continue to plan for the needs of an aging society, these findings suggest that HCBS may provide important benefits beyond maintaining independence and dignity. Further research is still needed to understand how the type and amount of HCBS affects nursing home placement, mortality, and quality of life.

## Supplementary Information

**Additional file 1.**

## Data Availability

The datasets analyzed for this study were made available to the authors under a data use agreement with the Commonwealth of Pennsylvania Department of Human Services reside and are not publicly available due to privacy and confidentiality concerns.
