# Supplementary material for: Risk of hospitalization associated with different constellations of home & community based services
Source: BMC Geriatr. 2023 Jan 20;23:36. doi: 10.1186/s12877-022-03676-2 (PMC9862558; doi:10.1186/s12877-022-03676-2)
Supplement: Supplementary file 1 — Additional file 1. [file 12877_2022_3676_MOESM1_ESM.docx]

**Appendices**

Below are alternative specifications for the model shown in Table 3.

Appendix A presents is a logistic regression model that uses robust standard errors to examine the risk of hospitalization associated with different constellations of HCBS. This model however does not include people who had applied to receive Medicaid funded HCBS and were deemed ineligible. People receiving low levels of PAS were the reference category when evaluating risk of hospitalization.

Appendix B presents the predicted probabilities for this model.

Appendix C presents a Generalized Estimating Equation to evaluate the risk of hospitalization associated with different constellations of HCBS. This model does include community dwelling elderly people who are not receiving Medicaid funded HCBS.

| Appendix A |  |  |  |  |
| --- | --- | --- | --- | --- |
|  | Odds Ratio | Std. Err. | 95% Confidence Interval | |
| Gender (Male) |  |  |  |  |
| Female | 0.916 | 0.021 | 0.877 | 0.958 |
| Race (White) |  |  |  |  |
| Black, Non-Hispanic | 1.272 | 0.032 | 1.210 | 1.337 |
| Hispanic, White | 0.948 | 0.078 | 0.806 | 1.114 |
| Asian American | 0.682 | 0.031 | 0.624 | 0.745 |
| Other Race | 0.867 | 0.037 | 0.798 | 0.943 |
| Geographic (Urban) |  |  |  |  |
| Rural | 0.971 | 0.031 | 0.912 | 1.033 |
| Age Category (65-70) |  |  |  |  |
| 70<-75 | 1.001 | 0.032 | 0.940 | 1.066 |
| 75<-80 | 0.969 | 0.032 | 0.909 | 1.033 |
| 80<-85 | 0.987 | 0.033 | 0.926 | 1.053 |
| 85+ | 1.062 | 0.032 | 1.000 | 1.127 |
| CHC Phase (Southwestern PAS) |  |  |  |  |
| South Eastern PA | 0.714 | 0.024 | 0.669 | 0.762 |
| Northern and Central PA | 1.011 | 0.035 | 0.945 | 1.082 |
| Level of Disability |  |  |  |  |
| Continence | 1.016 | 0.003 | 1.009 | 1.022 |
| ADL | 1.038 | 0.006 | 1.027 | 1.050 |
| IADL | 1.092 | 0.008 | 1.078 | 1.107 |
| Alzheimer's or Related Dementia | 0.881 | 0.022 | 0.839 | 0.924 |
| Living Situation (Lives Alone) |  |  |  |  |
| Lives with an Adult Child | 0.925 | 0.025 | 0.877 | 0.976 |
| Lives Other Person | 1.207 | 0.042 | 1.127 | 1.293 |
| Lives with Spouse | 0.809 | 0.027 | 0.758 | 0.864 |
| Lives with Other Family | 0.917 | 0.030 | 0.860 | 0.978 |
| Time Observed in the Data | 1.014 | 0.003 | 1.007 | 1.020 |
| Constellations of Care (Only uses Low PAS) |  |  |  |  |
| Low PAS |  |  |  |  |
|  |  |  |  |  |
| PAS and Adult Day Care | 0.526 | 0.026 | 0.477 | 0.579 |
| PAS and Delivered Meals | 1.112 | 0.032 | 1.051 | 1.178 |
| PAS, Adult Day Care, and Delivered  Meals | 0.697 | 0.091 | 0.541 | 0.900 |
| Medium PAS |  |  |  |  |
| Only PAS | 0.702 | 0.019 | 0.666 | 0.739 |
| PAS and Adult Day Care | 0.577 | 0.040 | 0.504 | 0.662 |
| PAS and Delivered Meals | 0.749 | 0.030 | 0.693 | 0.809 |
| PAS, Adult Day Care, and Delivered  Meals | 0.809 | 0.137 | 0.581 | 1.126 |
| High PAS |  |  |  |  |
| Only PAS | 0.616 | 0.026 | 0.568 | 0.669 |
| PAS and Adult Day Care | 0.631 | 0.128 | 0.423 | 0.941 |
| PAS and Delivered Meals | 0.687 | 0.050 | 0.597 | 0.792 |
| PAS, Adult Day Care, and Delivered  Meals | 0.849 | 0.281 | 0.444 | 1.623 |
| Count of Chronic Conditions (0-1 diseases) |  |  |  |  |
| 2-3 | 0.993 | 0.033 | 0.930 | 1.061 |
| 4-5 | 1.416 | 0.047 | 1.327 | 1.511 |
| 6+ | 2.947 | 0.090 | 2.776 | 3.129 |
| Constant | 0.064 | 0.004 | 0.057 | 0.073 |

| Appendix B |  |  |  |  |
| --- | --- | --- | --- | --- |
|  | Predicted Risk of Hospitalization | Std. Err. | 95% Confidence Interval | |
| Constellations with Low PAS |  |  |  |  |
| Only PAS | 0.152 | 0.002 | 0.148 | 0.157 |
| PAS and Adult Day Care | 0.086 | 0.004 | 0.079 | 0.094 |
| PAS and Delivered Meals | 0.167 | 0.003 | 0.160 | 0.173 |
| PAS, Adult Day Care, and Delivered  Meals | 0.111 | 0.013 | 0.086 | 0.137 |
| Constellations with Medium PAS |  |  |  |  |
| Only PAS | 0.112 | 0.002 | 0.108 | 0.116 |
| PAS and Adult Day Care | 0.094 | 0.006 | 0.083 | 0.105 |
| PAS and Delivered Meals | 0.119 | 0.004 | 0.111 | 0.126 |
| PAS, Adult Day Care, and Delivered  Meals | 0.127 | 0.019 | 0.091 | 0.164 |
| Constellations with High PAS |  |  |  |  |
| Only PAS | 0.100 | 0.003 | 0.093 | 0.106 |
| PAS and Adult Day Care | 0.102 | 0.019 | 0.066 | 0.138 |
| PAS and Delivered Meals | 0.110 | 0.007 | 0.097 | 0.123 |
| PAS, Adult Day Care, and Delivered  Meals | 0.132 | 0.038 | 0.058 | 0.207 |

| Appendix C |  |  |  |  |
| --- | --- | --- | --- | --- |
|  | Coef. | P>z | 95% Confidence Interval | |
| Gender (Male) |  |  |  |  |
| Female | -0.146 | >0.001 | -0.19 | -0.10 |
| Race (White) |  |  |  |  |
| Black, Non-Hispanic | 0.232 | >0.001 | 0.17 | 0.29 |
| Hispanic, White | -0.037 | 0.68 | -0.21 | 0.14 |
| Asian American | -0.451 | >0.001 | -0.55 | -0.35 |
| Other Race | -0.160 | >0.001 | -0.25 | -0.07 |
| Geographic (Urban) |  |  |  |  |
| Rural | -0.042 | 0.213 | -0.11 | 0.02 |
| Age Category (65-70) |  |  |  |  |
| 70<-75 | -0.027 | 0.413 | -0.09 | 0.04 |
| 75<-80 | -0.064 | 0.066 | -0.13 | 0.00 |
| 80<-85 | -0.054 | 0.129 | -0.12 | 0.02 |
| 85+ | 0.012 | 0.717 | -0.05 | 0.08 |
| CHC Phase (Southwestern PAS) |  |  |  |  |
| South Eastern PA | -0.347 | >0.001 | -0.42 | -0.27 |
| Northern and Central PA | -0.019 | 0.587 | -0.09 | 0.05 |
| Level of Disability |  |  |  |  |
| Continence | 0.016 | >0.001 | 0.01 | 0.02 |
| ADL | 0.043 | >0.001 | 0.03 | 0.05 |
| IADL | 0.060 | >0.001 | 0.05 | 0.07 |
| Alzheimer's or Related Dementia | -0.108 | >0.001 | -0.16 | -0.05 |
| Living Situation (Lives Alone) |  |  |  |  |
| Lives with Child | -0.021 | 0.502 | -0.08 | 0.04 |
| Lives Other Person | 0.031 | 0.369 | -0.04 | 0.10 |
| Lives with Spouse | -0.184 | >0.001 | -0.26 | -0.11 |
| Lives with Other Family | -0.013 | 0.732 | -0.08 | 0.06 |
| Time Observed in the Data | 0.015 | >0.001 | 0.01 | 0.02 |
| Constellations of Care (NFI) |  |  |  |  |
| Low PAS |  |  |  |  |
| Only PAS | 0.210 | >0.001 | 0.15 | 0.27 |
| PAS and Adult Day Care | -0.484 | >0.001 | -0.60 | -0.36 |
| PAS and Delivered Meals | 0.304 | >0.001 | 0.23 | 0.38 |
| PAS, Adult Day Care, and Delivered Meals | -0.153 | 0.343 | -0.47 | 0.16 |
| Medium PAS |  |  |  |  |
| Only PAS | -0.140 | >0.001 | -0.21 | -0.07 |
| PAS and Adult Day Care | -0.366 | >0.001 | -0.54 | -0.19 |
| PAS and Delivered Meals | -0.093 | 0.065 | -0.19 | 0.01 |
| PAS, Adult Day Care, and Delivered Meals | -0.037 | 0.871 | -0.48 | 0.40 |
| High PAS |  |  |  |  |
| Only PAS | -0.235 | >0.001 | -0.34 | -0.13 |
| PAS and Adult Day Care | -0.207 | 0.341 | -0.63 | 0.22 |
| PAS and Delivered Meals | -0.138 | 0.108 | -0.31 | 0.03 |
| PAS, Adult Day Care, and Delivered Meals | 0.169 | 0.627 | -0.51 | 0.85 |
| Count of Chronic Conditions (0-1 diseases) |  |  |  |  |
| 2-3 | 0.127 | >0.001 | 0.06 | 0.20 |
| 4-5 | 0.493 | >0.001 | 0.43 | 0.56 |
| 6+ | 1.218 | >0.001 | 1.15 | 1.28 |
| Constant | -2.841 | >0.001 | -2.96 | -2.72 |
